# Autoimmune Factor V Deficiency That Took 16 Years to Diagnose due to Pseudodeficiency of Multiple Coagulation Factors

**DOI:** 10.1155/2021/4657501

**Published:** 2021-01-12

**Authors:** Takaaki Kato, Takaya Hanawa, Mea Asou, Tomohiko Asakawa, Hisashi Sakamaki, Makoto Araki

**Affiliations:** ^1^Department of Internal Medicine, Suwa Central Hospital, Chino, Nagano 391-8503, Japan; ^2^Hematology Division, Tokyo Metropolitan Cancer and Infectious Diseases Center, Komagome Hospital, Bunkyo, Tokyo, Japan

## Abstract

A 70-year-old man presented to our hospital with intramuscular hemorrhage in the right thigh. He had exhibited a tendency to bleed for the last 16 years and had visited several medical institutions, but no diagnosis had been made. Since the risk of sudden bleeding was assumed to be high due to his age, we decided to examine him in our department. A coagulation abnormality with prothrombin time-international normalized ratio (PT-INR) of 4.5 and activated partial thromboplastin time (aPTT) of 99.6 seconds was observed, but the platelet count, fibrinogen, and PIVKAII were within normal limits. Coagulation activities of factor V, VII, VIII, IX, X, XI, XII, and XIII were all reduced. Anti-factor VIII and IX antibodies which were measured by the Bethesda method, lupus anti-coagulant (diluted Russell snake venom time method) and anti-cardiolipin antibody were also positive. The results of these tests were comparable to those undertaken 15 years ago when they were scrutinized at the university hospital. We suspected the presence of anti-factor V antibodies because there was a dissociation between the thrombotest values measured and those calculated from the PT-INR. Moreover, cross-mixing test showed an immediate inhibitor pattern. Subsequently, factor V antibodies were confirmed by the immunoblot method and the diagnosis of autoimmune factor V deficiency was made. When factor V, which is downstream of the coagulation cascade, is inhibited, coagulation test using the one-stage clotting method shows a pseudolow value. Therefore, extensive abnormalities of coagulation factor activity and inhibitor assay should be interpreted with caution, and the presence of a high titer of factor V inhibitor should be considered.

## 1. Introduction

Autoimmune coagulation factor deficiency is a hemorrhagic disease caused by the appearance of autoantibodies to coagulation factors and inhibition of their bioactivity. Since a delay in diagnosis and treatment can be life-threatening, accurate and timely diagnosis and treatment are clinically important. Although there are numerous reports on acquired hemophilia caused by a deficiency of factor VIII, reports on other coagulation factor deficiencies are relatively rare.

Here, we report a confirmed case with autoimmune factor V (FV) deficiency diagnosed 16 years after the onset of symptoms, at the age of 70 years. Although the patient had symptoms of bleeding, he had reduced activity of many coagulation factors, which prevented us from identifying the cause of the disease earlier.

### 1.1. Case Presentation

A 70-year-old man presented to our hospital with complaint of right thigh pain. A computed tomography (CT) scan revealed a hematoma in the right thigh. We recommended hospitalization, but the patient wanted outpatient treatment with tranexamic acid because he had received the same treatment earlier for this condition. He received hemostatic infusion for three consecutive days in the outpatient clinic, and hemoglobin level of 13.2 g/dl from one month ago dropped to 11.3 g/dl on the day of injury and to 7.9 g/dl two days after the injury. However, there was no further progression of anemia and the pain decreased. Therefore, the infusion was stopped on the fourth day.

**Figure 1:**
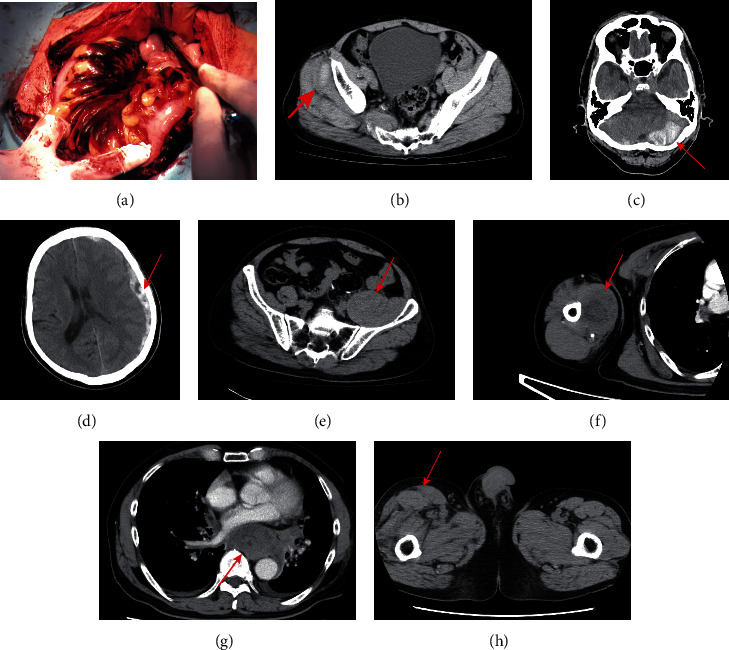
Major bleeding events that occurred during the course of the disease. 55 years old: intestinal hematoma (a). 59 years old: right glenohumeral and small pelvic cavity hematoma (b). 60 years old: left subacute subdural hematoma and small pelvic cavity hematoma (c). 60 years old: left acute subdural hematoma (d). 63 years old: intramuscular hematoma in the left iliopsoas muscle (e). 67 years old: right brachial intramuscular hematoma (f). 69 years old: intramediastinal hemorrhage (g). 70 years old: right intramuscular hematoma in the right thigh (h). (b)–(h) were light and managed with conservative treatment with tranexamic acid.

When we looked at the past medical records, he had presented to our hospital with the principal complaint of persistent subcutaneous hemorrhage at the age of 54 years. He had an abnormal prothrombin time (PT) % of 21.0% and activated partial thromboplastin time (aPTT) of 99.4 seconds. Consequently, he was referred to two university hospitals but no specific cause could be found. The doctor at the hospital had said, “factor VIII 4.7% and factor IX 0.6% suggest a broad spectrum of coagulation factor abnormalities. Although he had positive lupus anticoagulant (LAC), the diagnosis of antiphospholipid antibody syndrome could not be made. We cannot make a diagnosis, but FFP improves the coagulation activity to some extent, so use it in the event of bleeding.” Subsequently, there were numerous recurrent bleeding events, with major bleeding occurring every two years, such as in the ilium, pelvic cavity, subdural, iliopsoas, brachial muscles, and mediastinum (Figure 1). However, many of these bleeds healed spontaneously within a few days with conservative treatment. For this reason, the patient was given the provisional diagnosis of “circulating anticoagulant factor disorder” at the outpatient clinic of our hospital. Considering the age of the patient, the possibility of additional serious bleeding events was high, and therefore, we decided to examine the patient again.

He had never undergone a gastroscopy or surgery until the age of 54. He had no history of chronic diseases, such as allergic diseases and collagen diseases. His only regular medication was tranexamic acid. There was no subcutaneous hemorrhaging on physical examination. Blood tests showed a prolonged PT-INR of 4.5, along with aPTT of 99.6 seconds (Table 1). As reported 16 years ago, coagulation activities of factor V, VII, VIII, IX, X, XI, XII, and XIII were all reduced (1–26.5%). In addition, lupus anticoagulant (diluted Russell snake venom time method) and cardiolipin antibodies were positive, as were anti-factor VIII and IX antibodies by the Bethesda method.

Both PT-INR and aPTT were prolonged, which led us to suspect diseases such as disseminated intravascular coagulation (DIC), liver failure, antiphospholipid antibody syndrome, vitamin K deficiency (including due to warfarin), and drugs (heparin, argatroban, and DOAC). However, platelet count, Fib, and proteins induced by vitamin K antagonism (PIVKA) test were within the normal range, and the disease course did not fit these diagnoses.

Therefore, we suspected a deficiency of coagulation factor II, V, and X which are common to the coagulation pathways evaluated by PT and aPTT. We first performed the hepaplastin test (HPT) and thrombotest (TT) to investigate whether the coagulation tests changed with factor V (FV) supplementation (Table 1). The TT value was 3.7%, while the TT value calculated from PT-INR was 21% [1]. We determined that the patient was FV deficient because of the difference in TT induced by a high-dose of bovine FV supplementation.

**Figure 2:**
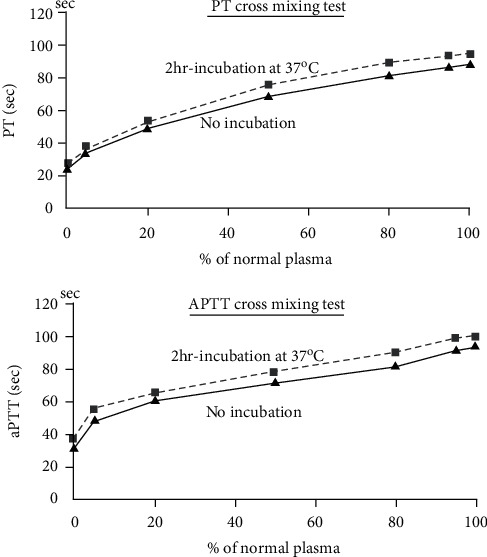
Cross-mixing test.

Next, we performed a cross-mixing test to determine whether FV deficiency was congenital or acquired. The results showed an immediate type of inhibitory pattern (Figure 2). Incubation at 37°C for 2 hours is required for anti-factor VIII antibody because of its weak inhibitory effect, whereas anti-FV antibody has a strong binding power and does not require incubation. This case also showed an immediate inhibitory response, suggesting the presence of anti-FV antibodies.

Since anti-FV antibody measurement is a laboratory-level test, we asked other institutions to measure them. As expected, both an immunoblot assay and enzyme-linked immunosorbent assay (ELISA) using purified human plasma FV confirmed the presence of anti-FV autoantibodies [2] and he was diagnosed with autoimmune FV deficiency. The plan is to treat him with immunosuppressive drugs in the future.

## 2. Discussion

FV deficiency is a rare disease and is known to cause major bleeding in some cases, but there are no reported cases with long-term untreated follow-up. Since this case had not been diagnosed earlier, therefore, we were able to obtain the clinical history for the last 16 years. The main feature of this case was that the correct diagnosis could not be made due to the extensive abnormalities found in the coagulation factor activity and inhibitor assays. The underlying reason is that the phenomenon of “pseudodeficiency of multiple coagulation factors” is not well known.

The prevalence of autoimmune FV deficiency is extremely low, at 0.09 cases per million individuals per year, compared with 1.48 cases with autoimmune FVIII deficiency [3, 4]. Primary symptoms differ from hemophilia A in that the majority of the primary symptoms are urologic, gastrointestinal, and respiratory bleeding, and there is little intra-articular bleeding [4, 5]. While usually asymptomatic, 12–17% patients may have fatal bleeding [6]. Most cases are idiopathic, but some cases may occur with cancer or collagen disease in the background or after the use of surgical hemostatic agents (e.g., bovine-derived thrombin products) [7]. This case was considered idiopathic because there was no history of previous gastroscopy or surgery. Normally no treatment is necessary. However, in the event of bleeding, treatment with FV supplementation is used as a coping strategy. Although fresh frozen plasma (FFP) is often used, platelet transfusion is also considered useful because of the abundance of coagulation FV in platelet alpha granules.

This case was characterized by the phenomenon of “pseudodeficiency of multiple coagulation factors” [2, 8], and knowledge of the one-stage clotting method, which is also performed in this case, is essential to understand this phenomenon (Suppl.  Figure 1). The one-stage clotting method is a PT or aPTT-based assay that is widely used for the measurement of coagulation factor activity and inhibitors due to its simplicity and automation of the measurement system. The coagulation factor activity assay is performed by mixing the target factor-deficient plasma with the patient's plasma, and the coagulation inhibitor assay is performed by mixing the patient's plasma with normal plasma, and the resulting plasma is used to measure PT or aPTT. In other words, these assays do not measure the activity of a coagulation factor itself, but rather perform an indirect search for the activity of a coagulation factor by measuring the time at which fibrin is formed (clotting time). Therefore, this assay system cannot detect unexpected abnormalities such as anti-FV antibodies as in our case. In such cases, the clotting time is always prolonged by any coagulation factor activity or inhibitor assay because FV activity, which is downstream of the coagulation cascade, is inhibited by the anti-FV antibody. Dilute Russell's viper venom time (dRVVT) is always an outlier for the same reason. To prevent “pseudodeficiency of multiple coagulation factors,” the two-step coagulation method, the chromogenic substrate method, and the enzyme-linked immunosorbent (ELISA) method are available, but they are not widely used because of their complexity.

The diagnosis in this case was made by a traditional assay called TT and HPT (Suppl.  Figure 2). This assay confirms the activity of vitamin K-dependent coagulation factors by adding the coagulation factor other than II, VII, and X (created by absorbing the vitamin K-dependent coagulation factors with barium sulfate) and the activator to patient plasma. The activator is thromboplastin, differentiated by the fact that TT is bovine and HPT is of rabbit origin. The usefulness of this test for determining FV deficiency has been reported in the past [9].

Furthermore, the present case was positive for cardiolipin antibodies, which are also found in other cases of acquired FV inhibition, suggesting a common mechanism [10]. The assay system for detecting cardiolipin antibodies is the indirect ELISA (Suppl.  Figure 3). The indirect ELISA measures not only anti-cardiolipin antibodies that bind to cardiolipin but also antibodies to plasma proteins that bind to cardiolipin [11, 12]. Since FV is known to attach to phospholipids via the C2 domain [13], this finding should not indicate the presence of antiphospholipid syndrome (APS) and is considered false positive. However, this is only conjecture and essentially requires direct proof by immunoprecipitation.

In conclusion, we have described a case of autoimmune FV deficiency that went undiagnosed for 16 years due to “pseudodeficiency of multiple coagulation factors.” Autoimmune FV deficiency requires early diagnosis and treatment because of the life-threatening bleeding that may occur once every two years. When, as in the present case, extensive abnormalities of the coagulation factor activity and inhibitor assays are found, the presence of a high titer of FV inhibitor should be considered and the laboratory values should be interpreted carefully. In such a case, diagnosis using TT and HPT is considered useful.

Both PT and aPTT showed an inhibitor pattern. In addition, unlike anti-factor VIII antibody, there was little change after 2 hours of incubation because it showed an immediate type of reaction.

## Figures and Tables

**Table 1:** The main blood test of coagulation system at the university hospital 15 years ago and this visit.

	15 years ago	This visit	Units	Normal value
PT-INR	3.1	4.54		0.91～1.12
aPTT	120	99.6	sec	25～40
Fib	335	228.7	mg/dL	200～400
FDP	4.7	2.5	*μ*mg/mL	0～5
D-dimer	1.44	0.7	*μ*mg/mL	0～1
Antithrombin III	108.4	113.7	%	80～130
Thrombotest	16.4	21.2	%	70.0～
Hepaplastin test	33.2	36.9	%	70.0～130
Plasminogen	106.3	112	%	80～130
TAT		1.7	ng/mL	<4.0
PIC		0.7	*μ*mg/mL	<0.8
TM		3	FU/mL	2.1～4.1
Protein C activity	86.8	118	%	70～140
Free protein S antigen	120	94.2	%	60～150
ANA		×40 (SPECKL)		<40
Anti-CL-*β*2GPI Ab		＜1.3	U/mL	<3.5
Anti-cardiolipin Ab		46	U/mL	<10
Lupus anticoagulant				
(Diluted Russell's viper venom time)		(Not coagulate)	(Nomalized ratio)	<1.2
Lupus anticoagulant				
(Silica clotting time)		4.6	sec	
(Difference time)	＜8			
Factor V activity	17	12.4	%	73～122
Factor VII activity	28	26.5	%	54～162
Factor VIII activity	3.4	5.3	%	78～165
Factor IX activity	1.1	<1.0	%	67～152
Factor X activity	25	24.5	%	58～200
Factor XI activity	3	<1.0	%	75～137
Factor XII activity	23	11.2	%	36～152
Factor XIII activity	123	109	%	70～140
PIVKA-II		32	mAU/mL	0～40
Factor VIII inhibitor		3.8	BU/ml	<1.0
Factor IX inhibitor		3.5	BU/ml	<1.0

Ab, antibody; TAT, thrombin antithrombin III complex; PIC, plasmin-*α*2 plasmin inhibitor complex; TM, thrombomodulin; anti-*β*2GPI, anti-*β*2 glycoprotein I; PIVKAII, protein induced by vitamin K absence II.

## Data Availability

Data of the case report are available upon request to the corresponding author via mail.

## Supplementary Materials

Supplementary Materials Supplementary Figure 1: mechanism of pseudodeficiency of multiple coagulation factors. The coagulation factor activity assay and coagulation inhibitor assay (Bethesda assay) are usually performed using a one-stage clotting method. Specifically, the coagulation factor activity assay is performed by mixing the target factor-deficient plasma with the patient's plasma, and the coagulation inhibitor assay is performed by mixing the patient's plasma with normal plasma, and this plasma is subsequently used to measure PT or aPTT. The diluted Russell's viper venom time (dRVVT) is a test that directly stimulates factor X, that is, it eliminates the upstream effects, allowing one-stage clotting method to measure the effects of antibodies to phospholipids (PL). None of these tests measure the activity or inhibitors of the clotting factors themselves. These tests indirectly measure the activity or inhibitors of the clotting factors by measuring the time of fibrin formation (clotting time). In such a case, clotting time is always prolonged regardless of which coagulation factor activity and inhibitor assay are used, because FV activity downstream of the coagulation cascade is inhibited by the anti-FV antibodies (pseudodeficiency of multiple coagulation factors). Supplementary Figure 2: thrombotest and hepaplastin test. TT and HPT are an assay using the one-stage clotting method. This assay confirms the activity of vitamin K-dependent coagulation factors by adding the activator and plasma to barium sulfate which absorbs the vitamin K coagulation factors (that is, coagulation factors other than II, VII, and X). In the present case, large amounts of FV were supplemented to neutralize the anti-FV antibodies, resulting in a dissociation from PT time. Supplementary Figure 3: false cardiolipin antibodies. Measurement of cardiolipin antibodies was performed using ELISA (left figure). If antibodies are present against the protein that attaches to cardiolipin, the test is positive (right figure, pseudocardiolipin antibody).
