# Supplementary figures and images for: Autoimmune Factor V Deficiency That Took 16 Years to Diagnose due to Pseudodeficiency of Multiple Coagulation Factors
Source: Case Rep Med. 2021 Jan 12;2021:4657501. doi: 10.1155/2021/4657501 (PMC7815411; doi:10.1155/2021/4657501)

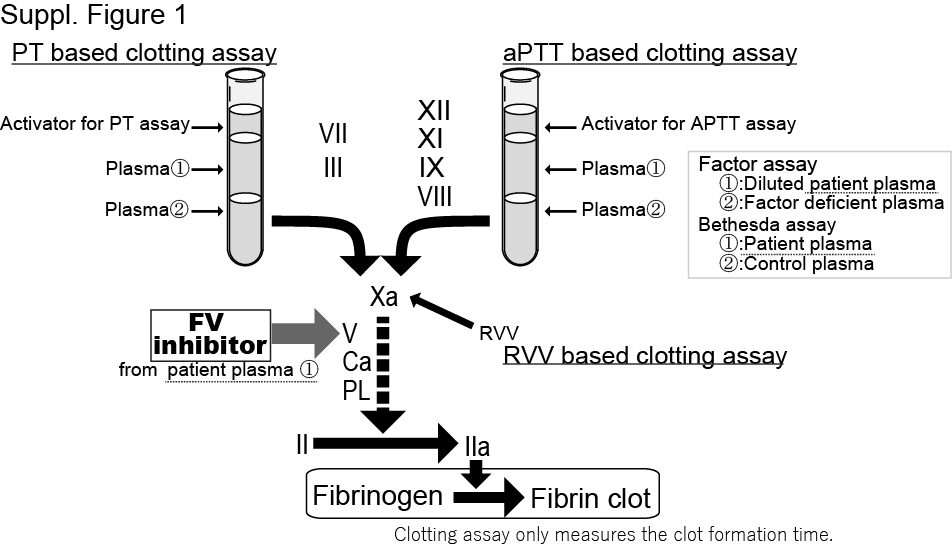

Supplement: Supplementary Materials — Supplementary Figure 1: mechanism of pseudodeficiency of multiple coagulation factors. The coagulation factor activity assay and coagulation inhibitor assay (Bethesda assay) are usually performed using a one-stage clotting method. Specifically, the coagulation factor activity assay is performed by mixing the target factor-deficient plasma with the patient's plasma, and the coagulation inhibitor assay is performed by mixing the patient's plasma with normal plasma, and this plasma is subsequently used to measure PT or aPTT. The diluted Russell's viper venom time (dRVVT) is a test that directly stimulates factor X, that is, it eliminates the upstream effects, allowing one-stage clotting method to measure the effects of antibodies to phospholipids (PL). None of these tests measure the activity or inhibitors of the clotting factors themselves. These tests indirectly measure the activity or inhibitors of the clotting factors by measuring the time of fibrin formation (clotting time). In such a case, clotting time is always prolonged regardless of which coagulation factor activity and inhibitor assay are used, because FV activity downstream of the coagulation cascade is inhibited by the anti-FV antibodies (pseudodeficiency of multiple coagulation factors). Supplementary Figure 2: thrombotest and hepaplastin test. TT and HPT are an assay using the one-stage clotting method. This assay confirms the activity of vitamin K-dependent coagulation factors by adding the activator and plasma to barium sulfate which absorbs the vitamin K coagulation factors (that is, coagulation factors other than II, VII, and X). In the present case, large amounts of FV were supplemented to neutralize the anti-FV antibodies, resulting in a dissociation from PT time. Supplementary Figure 3: false cardiolipin antibodies. Measurement of cardiolipin antibodies was performed using ELISA (left figure). If antibodies are present against the protein that attaches to cardiolipin, the test is positive (right f [file 4657501.f1.zip › 4657501.f1/Suppl. Figure 1 (1).jpg]

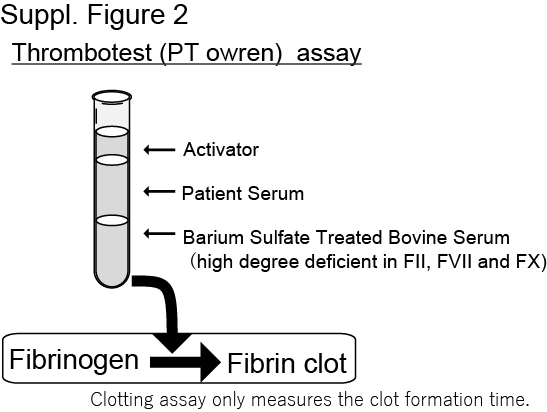

Supplement: Supplementary Materials — Supplementary Figure 1: mechanism of pseudodeficiency of multiple coagulation factors. The coagulation factor activity assay and coagulation inhibitor assay (Bethesda assay) are usually performed using a one-stage clotting method. Specifically, the coagulation factor activity assay is performed by mixing the target factor-deficient plasma with the patient's plasma, and the coagulation inhibitor assay is performed by mixing the patient's plasma with normal plasma, and this plasma is subsequently used to measure PT or aPTT. The diluted Russell's viper venom time (dRVVT) is a test that directly stimulates factor X, that is, it eliminates the upstream effects, allowing one-stage clotting method to measure the effects of antibodies to phospholipids (PL). None of these tests measure the activity or inhibitors of the clotting factors themselves. These tests indirectly measure the activity or inhibitors of the clotting factors by measuring the time of fibrin formation (clotting time). In such a case, clotting time is always prolonged regardless of which coagulation factor activity and inhibitor assay are used, because FV activity downstream of the coagulation cascade is inhibited by the anti-FV antibodies (pseudodeficiency of multiple coagulation factors). Supplementary Figure 2: thrombotest and hepaplastin test. TT and HPT are an assay using the one-stage clotting method. This assay confirms the activity of vitamin K-dependent coagulation factors by adding the activator and plasma to barium sulfate which absorbs the vitamin K coagulation factors (that is, coagulation factors other than II, VII, and X). In the present case, large amounts of FV were supplemented to neutralize the anti-FV antibodies, resulting in a dissociation from PT time. Supplementary Figure 3: false cardiolipin antibodies. Measurement of cardiolipin antibodies was performed using ELISA (left figure). If antibodies are present against the protein that attaches to cardiolipin, the test is positive (right f [file 4657501.f1.zip › 4657501.f1/Suppl. Figure 2 (1).jpg]

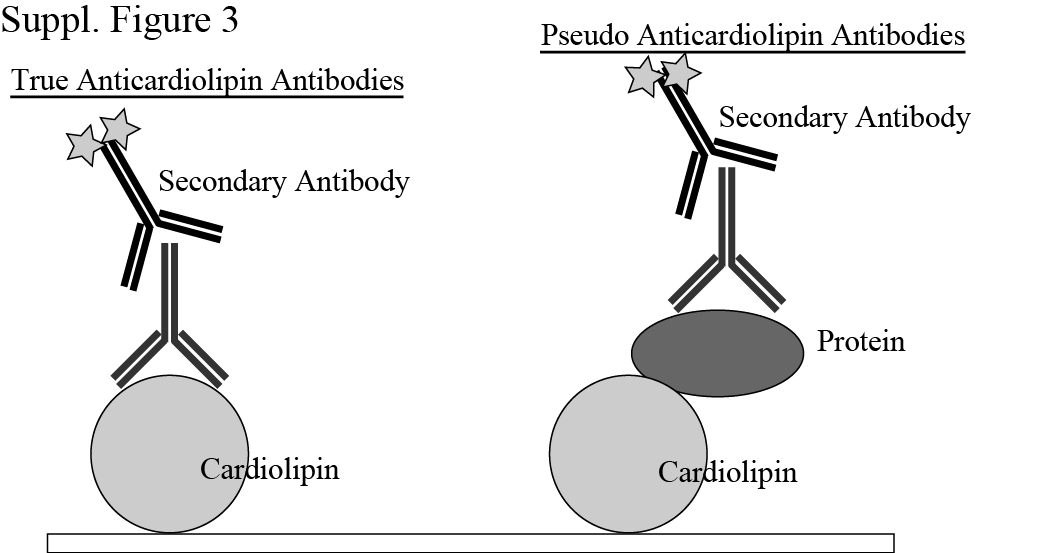

Supplement: Supplementary Materials — Supplementary Figure 1: mechanism of pseudodeficiency of multiple coagulation factors. The coagulation factor activity assay and coagulation inhibitor assay (Bethesda assay) are usually performed using a one-stage clotting method. Specifically, the coagulation factor activity assay is performed by mixing the target factor-deficient plasma with the patient's plasma, and the coagulation inhibitor assay is performed by mixing the patient's plasma with normal plasma, and this plasma is subsequently used to measure PT or aPTT. The diluted Russell's viper venom time (dRVVT) is a test that directly stimulates factor X, that is, it eliminates the upstream effects, allowing one-stage clotting method to measure the effects of antibodies to phospholipids (PL). None of these tests measure the activity or inhibitors of the clotting factors themselves. These tests indirectly measure the activity or inhibitors of the clotting factors by measuring the time of fibrin formation (clotting time). In such a case, clotting time is always prolonged regardless of which coagulation factor activity and inhibitor assay are used, because FV activity downstream of the coagulation cascade is inhibited by the anti-FV antibodies (pseudodeficiency of multiple coagulation factors). Supplementary Figure 2: thrombotest and hepaplastin test. TT and HPT are an assay using the one-stage clotting method. This assay confirms the activity of vitamin K-dependent coagulation factors by adding the activator and plasma to barium sulfate which absorbs the vitamin K coagulation factors (that is, coagulation factors other than II, VII, and X). In the present case, large amounts of FV were supplemented to neutralize the anti-FV antibodies, resulting in a dissociation from PT time. Supplementary Figure 3: false cardiolipin antibodies. Measurement of cardiolipin antibodies was performed using ELISA (left figure). If antibodies are present against the protein that attaches to cardiolipin, the test is positive (right f [file 4657501.f1.zip › 4657501.f1/Suppl. Figure 3 (1).jpg]
